# Can depth-based restrictions avoid bird bycatch while maintaining catch rates in the Icelandic lumpfish fishery? A comment on Rouxel *et al*. (2023)

**DOI:** 10.1098/rsos.231981

**Published:** 2024-07-24

**Authors:** J. Kennedy, G. M. Sigurdsson

**Affiliations:** ^1^ Marine and Freshwater Research Institute, Árnagötu 2-4, 400 Ísafjörður, Iceland; ^2^ Marine and Freshwater Research Institute, Fornubúðum 5, 220 Hafnarfjörður, Iceland

**Keywords:** depth-based restrictions, bird, bycatch, *Cyclopterus lumpus*, gillnet, Iceland

*R. Soc. Open Sci.* **10**: 230783 (Published online 25 October 2023) (https://doi.org/10.1098/rsos.230783)

The bycatch of birds in bottom set gillnets in Iceland is recognized as an issue, with several technical solutions being proposed and trialled, but so far, without success [1,2]. Using data collected during an investigation on whether ‘looming eyes’ buoys could reduce bycatch in lumpfish (*Cyclopterus lumpus*) gillnets, Rouxel *et al*. [1] proposed ‘that limiting fishing to waters more than 50 m deep could save between 5000 and 9300 seabirds every year, arrest the population decline of endangered black guillemots (*Cepphus grylle*) in Iceland, while having only a marginal effect on target fish catch’. This is an interesting result and if this is indeed the case has potential to improve the environmental impact of this fishery. The study by Rouxel *et al*. [1] was based upon the catches of 84 fishing trips from 7 vessels, of which 21 fishing trips from 3 fishing vessels fished at a depth >50 m, with one boat accounting for 10 of these trips. The study took place in Húnaflói Bay, in northern Iceland (figure 1). The fishery in this bay accounts for an average of 17% of the total lumpfish landings in Iceland.

**Figure 1.**
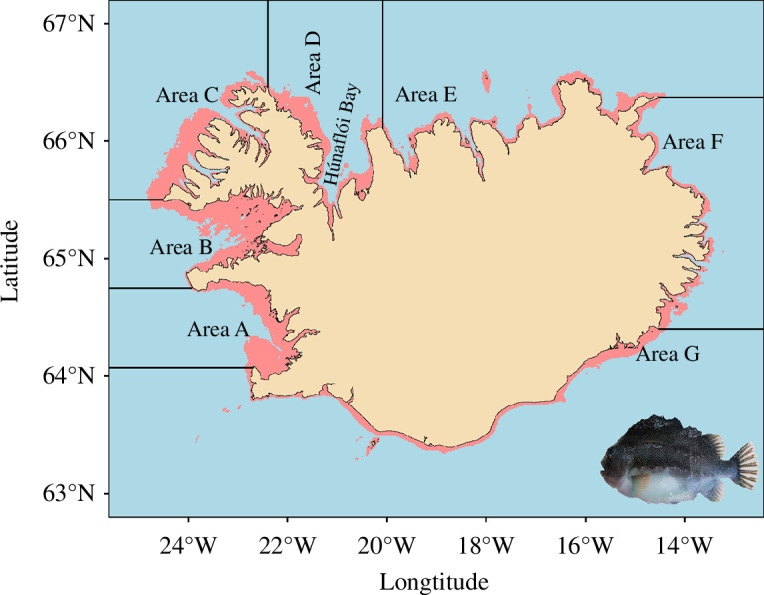
Map of Iceland showing lumpfish management areas and location of Húnaflói Bay. Coastal areas of <50 m depth is shown in red (depth data from NOAA via marmap [3]).

The proposal by Rouxel *et al.* [1] needs to be carefully examined as large reductions in catch could result in significant economic loss for fishers. Most of the vessels participating in the Icelandic lumpfish fishery are <15 gt and based in small communities, so regulations which severely disrupt the fishery can have major socio-economic consequences. The aim of this study is to explicitly examine the proposal made by Rouxel *et al.* [1] using a larger dataset which covers a larger area and greater number of fishing vessels. The proposal from Rouxel *et al* [1] can be broken down into two components:

— Can limiting the fishery to depths deeper than 50 m significantly reduce bycatch of birds in the Icelandic gillnet fishery for lumpfish?
— Will implementing a depth-based limitation have only a marginal effect on target catch?

Examining component one, seabird bycatch, data from onboard inspections from the Directorate of Fisheries collected between 2014 and 2022 in all management regions was used. Analyses of these data show that while seabird bycatch of many species decreases with depth, it is highest between 0–10 and 40–50 m depth (table 1). That trend is driven by large catches of eider ducks (*Somateria mollissima*) and black guillemots (*Cepphus grylle*) in the shallowest depth band and catches of common guillemots (*Uria aalge*) at deeper depth that are caught in significantly higher numbers at 40–50 m depth. Common guillemots are also sometimes caught in large numbers in the gillnet fishery for cod, at depths exceeding 50 m depth [4]. These data suggest that while bycatch of some species might be reduced with such depth-based restrictions, it might increase bycatch of other species that dive to greater depths.

**Table 1.** Observed seabird bycatch rate (*n*/fishing trips) by depth band species in onboard inspections conducted by the Directorate of Fisheries between 2014 and 2022. The four most common species are shown separately (98% of total bycatch), as well as total bycatch. The cormorant category includes two species (*Phalacrocorax aristotelis* and *Phalacrocorax carbo*) owing to difficulties in species identification.

depth (m)	Eider ducks	common guillemots	black guillemots	cormorants	all seabirds
0–10	2.91	0.26	1.58	0.55	5.36
11–20	1.80	0.35	0.75	0.21	3.19
21–30	0.67	0.65	0.41	0.22	2.01
31–40	0.23	0.34	0.21	0.07	0.91
41–50	0.07	4.22	0.11	0.07	4.52
51+	0.39	0.90	0.02	0.04	1.42

We examined component two, marginal impact on target catches, using logbooks from the fishery. Every fishing boat in Iceland is required to submit a logbook each year which gives details of their fishing operations. Since 1980, between 173 and 447 boats have participated annually in the Icelandic lumpfish fishery [5]. Using these logbooks from 1980 to 2015, which contains information on 2289 vessels and approx. 228 000 days in which nets were hauled, we examined the depth distribution of the catch for each of the seven lumpfish management areas in Iceland (figure 1) and catch per unit effort (CPUE) (catch weight of lumpfish per net) versus depth. We used the logbooks from 1980 to 2015 as they had a consistent format. The logbooks from 2016 to 2022 were not included as there had been several changes in the format over this time, but there is no indication from these logbooks that there was any change in the fishery that would affect the results.

Data from logbooks show that for areas A–C, <1% of the catch came from depths >50 m and 3.3% for area G (figure 2), these four areas account for an average of 40% of the catch between 2008 and 2022. For areas D–F, 17, 12% and 22% of the catch came from depths >50 m. It is interesting to note where the 50 m contour lies in relation to the coastline, and the percentage of catches below 50 m depth. For areas A–C, the distance is much greater than for D–F. Regarding CPUE, for areas A–C and G, there was a significant (linear regression: *p* < 0.05) but weak correlation between depth and CPUE for depths 0–40 m which was positive for areas A and B and negative for areas C and G (figure 3). Owing to low fishing effort at depths >50 m, it was not possible to assess if catch rates could be maintained at depths >50 m. For areas D and E, CPUE was slightly lower at depths >50 m while in area F, CPUE was slightly higher at depths >50 m.

**Figure 2.**
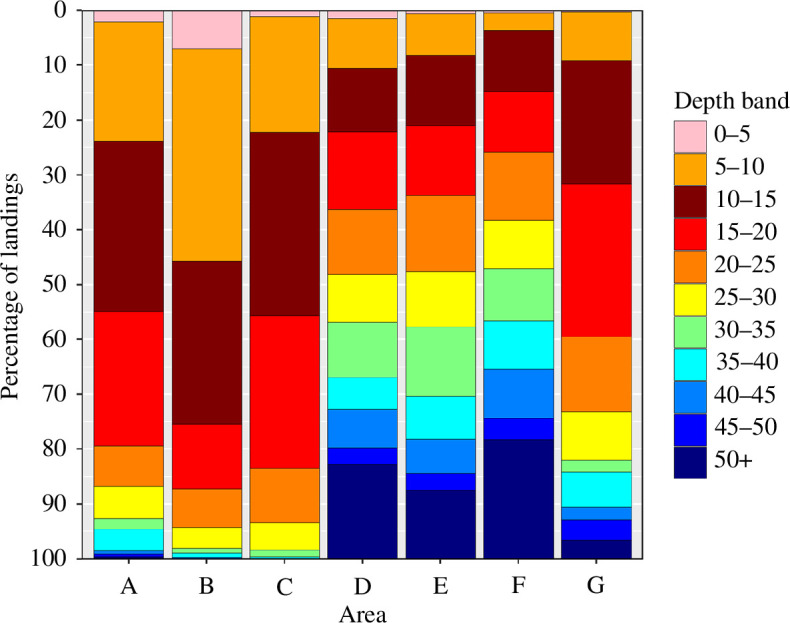
Percentage of landings from 1980 to 2015 by 5 m depth band as given in fishers logbooks.

**Figure 3.**
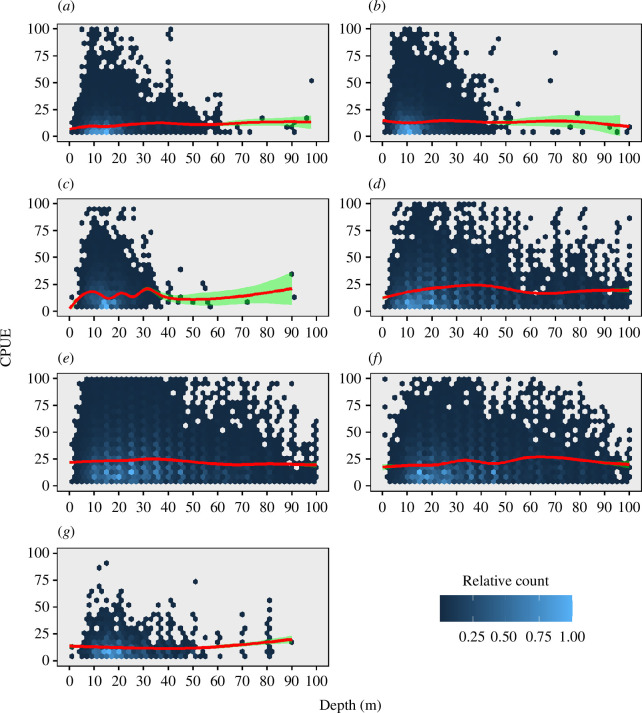
Hexagonal heatmap of CPUE versus depth in the Icelandic lumpfish gillnet fishery in each management area 1980–2015. Colour denotes the relative distribution of values within each management unit. Locally estimated scatterplot smoothing (LOESS) regression line (red) is shown together with standard error (green). CPUE values greater than 100 were excluded to aid visualization of the data.

The lumpfish fishery is a roe fishery and targets females as they come to coastal areas to spawn and selects for individuals close to spawning [6]. Lumpfish require a structured habitat for spawning with nests placed on areas of bedrock, boulders and vegetation and boulders, but do not site their nests in sandy areas [7–9]. This suggests that depth may not be an important factor *per se*, but the presence of suitable spawning areas, which are likely to be more common at depths <50 m and (or) closer to shore, are the primary driver of where lumpfish spawn. The primary substrate at depths >50 m for areas A–C is sand, sandy mud or muddy sand [10] which are unsuitable spawning habitats for lumpfish [7–9], suggesting catch rates are probably low beyond 50 m. North of Iceland, which encompasses areas D–F, the picture is more complex with rocky and hard substrate extending beyond 50 m, but this is restricted to specific sections of the coast. The presence of this hard substrate beyond 50 m may indicate why catch rates are maintained at this depth in these areas. However, depending on location, many fishers may not have access to suitable spawning/fishing areas at depths greater than 50 m given the heterogeneous nature of the seabed in these areas.

Where fishers place their nets is not random; many have been fishing in the same area for decades, and knowledge of fishing spots is passed down through generations. Thus, fishers will be placing their nets where there is known to be high catch rates and avoid areas where catch rates are low. Therefore, distribution of fishing locations is indicative of good fishing spots, and locations and depths where there is low fishing effort, would indicate poor fishing. With fishers continuing to fish in spots where there are good catches and abandoning spots of poor catches. The data presented in Rouxel *et al*. [1] and data from logbooks suggested that in certain areas, catch can be maintained at depths >50 m, but as Rouxel *et al*. [1] acknowledged, there is likely to be greater competition for these spots under a depth restricted scenario and these areas may not be able to accommodate the fishers that have been displaced. The data from logbooks suggest that in areas A–C and G, catch would not be able to be maintained if fishing was restricted to depths >50 m. Given the substrate at depths >50 m in areas A–C, it seems unlikely that there are suitable spawning areas for lumpfish and therefore unlikely to be suitable spots to fish for lumpfish. Even if there were suitable spots, a depth restriction of even depths >30 m would require the entire relocation of the fishery in these areas which would result in significant disruption to catch. It would probably take many years for fishers to locate good fishing spots comparable to their previous ones. In this regard, it seems very unlikely that a depth restriction of >50 m could have only a marginal effect on target catch.

When considering the information presented in the current study, it seems very likely that the effect of prohibiting fishing for lumpfish at depths >50 m, or even >30 m, would be a major decrease in catches and would probably end the fishery in several areas such as areas A and B (which would drastically cut bycatch). Given the heterogeneous nature of the seabed along the coast of Iceland and considering the distribution of common guillemot where mortality could increase under a depth restricted scenario, a more nuanced approach is likely to be required to reduce bycatch and maintain catch. This would require an examination of what measures would be effective in each specific area, and possibly at different locations within each area. We welcome the efforts by Rouxel *et al*. [1] to investigate novel solutions to the problem of bycatch and hope they will continue to investigate methods which could lower bycatch in gillnet fisheries, but it seems that simply limiting the fishery to depths >50 m is not going to be successful in reducing bycatch while maintaining catch.

## Data Availability

Logbooks used in the current study can be accessed at https://data.hafro.is/research/lumpfish_logbooks/.
